# Supplementary material for: Deletion of wfs1 Impairs Oligodendrocyte Precursor Cells Dorsal Distribution and Myelination Through the wfs1-hmgcs1 Axis in Zebrafish
Source: Biology (Basel). 2026 Aug 16;15(16):1402. doi: 10.3390/biology15161402 (PMC13510151; doi:10.3390/biology15161402)
Supplement: Supplementary file 1 [file biology-15-01402-s001.zip › File S1-Transcriptome sequencing data and differentially expressed genes (DEGs) in wfs1-- zebrafish at 5 dpf/2.KEGG/wfs1KOvsWT/UP/src/dre00250.html]

dre00250


- dre:334431

- Up regulated genes

334431\_1(1.27207238534102)

- dre:565235

- Up regulated genes

565235\_1(6.00315969790843)

- dre:447815

- Up regulated genes

447815(1.48422249409273)

- dre:447815

- Up regulated genes

447815(1.48422249409273)

Close
